# Immobilized Keratin HPLC Stationary Phase—A Forgotten Model of Transdermal Absorption: To What Molecular and Biological Properties Is It Relevant?

**DOI:** 10.3390/pharmaceutics15041172

**Published:** 2023-04-07

**Authors:** Anna Weronika Sobańska, Elżbieta Brzezińska

**Affiliations:** Department of Analytical Chemistry, Faculty of Pharmacy, Medical University of Lodz, ul. Muszyńskiego 1, 90-151 Lodz, Poland

**Keywords:** biomimetic chromatography, immobilized keratin stationary phase, immobilized artificial membrane chromatography, skin permeability, bioconcentration factor

## Abstract

Chromatographic retention data collected on immobilized keratin (KER) or immobilized artificial membrane (IAM) stationary phases were used to predict skin permeability coefficient (log ***K***_p_) and bioconcentration factor (log ***BCF***) of structurally unrelated compounds. Models of both properties contained, apart from chromatographic descriptors, calculated physico-chemical parameters. The log ***K***_p_ model, containing keratin-based retention factor, has slightly better statistical parameters and is in a better agreement with experimental log ***K***_p_ data than the model derived from IAM chromatography; both models are applicable primarily to non-ionized compounds.Based on the multiple linear regression (MLR) analyses conducted in this study, it was concluded that immobilized keratin chromatographic support is a moderately useful tool for skin permeability assessment.However, chromatography on immobilized keratin may also be of use for a different purpose—in studies of compounds’ bioconcentration in aquatic organisms.

## 1. Introduction

Many chemicals enter the human body through the skin. Transdermal absorption is an important route of drugs’ administration, and it is also very important in the context of environmental toxicology, since undesired xenobiotics are often absorbed transdermally. The skin permeability coefficient ***K***_p_ is defined according to Equation (1):

(1) $$K_{p}=\frac{K_{m}D}{h}$$

where: ***K***_m_—the partition coefficient between the stratum corneum and the vehicle; ***D***—the effective compound’s diffusion coefficient through the stratum corneum; ***h***—the diffusional pathlength.

The experimental values of skin permeability coefficients are measured in vivo (on human volunteers), ex vivo (on excised human skin), or on animal models [1], but such data are difficult to obtain due to ethical and financial problems, and the results of experiments in this area are often inconsistent due to variations in properties of different skin samples, even taken from the same human or animal.

Apart from skin absorption, an important property of compounds of environmental concern is their bioconcentration factor in aquatic organisms (***BCF***). The bioconcentration factor is the ratio of the chemical concentration in the organism (***C***_B_) and water (***C***_w_), accounting for the absorption via the respiratory route (e.g., gills) and skin. It is used to assess the bioaccumulation potential of compounds [2], especially in the absence of their bioaccumulation factor (***BAF***),which accounts for dietary, dermal, and respiratory exposures. According to different regulatory agencies, different criteria of bioaccumulation apply: bioaccumulative compounds have ***BCF*** > 5000 or ***BCF*** > 2000 [3]. In the absence of ***BAF*** or ***BCF*** data, lipophilicity measured as the octanol-water partition coefficient ***K***_ow_ is used to assess the compounds’ ability to bioaccumulate; if this is the case, the log ***K***_ow_ threshold for bioaccumulative compounds is 5 [3,4], 4.5 [5], or 3.3 [6]. Measured and estimated bioaccumulation data are also used to assign chemicals to three bioaccumulation categories: not significantly bioaccumulative (***BCF*** or ***BAF*** < 1000), bioaccumulative (***BCF*** or ***BAF*** between 1000 and 5000), and highly bioaccumulative (***BCF*** or ***BAF*** > 5000) [7].

The ethical and financial problems related to ***BCF*** determination are similar to those encountered during ***K***_p_ measurements. In in vivo experiments, the need to use human volunteers or lab animals, as well as the experiment timing, are the main limitations, and, for this reason, both ***K***_p_ and ***BCF*** are frequently assessed in vitro (using cell/tissue assays or non-cell models based on chromatography or electrochromatography) or in silico (calculations that can provide valuable information even without the access to compounds’ samples) [8,9,10].

Biomimetic chromatography essentially involves the application of stationary phases, containing proteins or phospholipids, or mobile phases, including micelles or microemulsions [11,12,13,14]. The components of biomimetic chromatographic systems (stationary or mobile phases) are designed to mimic some elements or functions of biomembranes and the interactions between these components and studied molecules resemble transport and partition phenomena encountered in a living being.

Immobilized artificial membrane (IAM) chromatography, with stationary phases containing adsorbed or covalently bound phosphatidylcholine (or, more recently, sphyngomyelin) groups, is used in modern lipohilicity studies, as well as in investigations of compounds’ affinity for phospholipids, related to many biological properties of solutes [15,16,17]. Chromatography on immobilized protein stationary phases was originally developed to separate enantiomers [18]; apart from that, some protein-based stationary phases simulate the interaction between a molecule and main plasma proteins, such as human serum albumin (HSA) [19,20,21,22] or α_1_ acid glycoprotein (AGP) [21,23,24,25]. Retention data obtained from chromatography in biomimetic systems are used to predict ADME (absorption, distribution, metabolism, and excretion) properties of compounds in early drug discovery phases [11,26], as well as their environmental impact—mobility in soil, bioconcentration/bioaccumulation, or aquatic toxicity [27,28,29,30,31]. Elements of natural biomembranes, incorporated in chromatographic systems used in pharmacokinetic studies, include also cholesterol or amide moieties [32,33].

Chromatographic descriptors have been used in skin permeability studies for many years, and separation (chromatographic or electrochromatographic) techniques used in these studies are liquid chromatography (HPLC or TLC), biopartitioning micellar chromatography, micellar electrokinetic chromatography, liposome electrokinetic chromatography, and two-dimensional gas chromatography (GC × GC) [34,35,36,37,38,39,40,41,42,43,44,45,46,47].

The relationships between the IAM chromatographic retention factor (***k****_IAM_*) and the skin permeability coefficient have been studied most frequently for small groups of compounds (*n* = 10 to 32), and the resulting dependencies are mostly univariate (linear or quadratic) [35,36,39,41], the exceptions being the studies in which additional variables, e.g., McGowan’s characteristic volume ***V*** or the octanol-water partition coefficient log ***K***_ow_ [33,35,36] were incorporated. In our earlier study [48] conducted for a large group of structurally unrelated compounds (*n* = 160), we demonstrated that log ***k***_IAM_ accounts for ca. 46% of total log ***K***_p_ variability, and the parameters whose contribution to log ***K***_p_ predictions is also significant are polar surface area (***PSA***) or polarizability (***α***).

Bioconcentration of compounds in aquatic organisms can be studied in vitro using descriptors derived from HPLC chromatography on C_18_, C_8_, C_2_, and phenyl-bonded silica sorbents (aromatic hydrocarbons [49]), C_18_ and cyanopropyl- and phenyl-bonded silica (aromatic hydrocarbons, alkylbenzenes, chlorinated benzenes, phthalates, nitroaromatics, phenols, and aniline [50]), and RP-18 TLC (organic sunscreens and cosmetic preservatives [51]).

More recently, the bioconcentration of compounds in aquatic organisms has been investigated using chromatography on IAM stationary phases, developed initially to mimic molecule–biomembrane interactions in ADME studies [31,52]. Earlier research pointed to the importance of additional parameters, incorporated alongside log ***k***_IAM_: (i) a biodegradation estimate, ***BioWin5***, calculated using the EPISuite^TM^ software and (to a lesser extent) topological polar surface area (***TPSA***) [52]; (ii) ***TPSA***—the fraction of sp^3^ carbon atoms (***F***_Csp3_) and hydrogen bond donor count (#***HD***) [31].

Turowski and Kaliszan postulated that predicting skin permeability of compounds should be based on molecules’ lipophilicity and interactions with keratin, which is an important constituent of the outmost layer of the epidermis [34]. An immobilized keratin-based stationary phase, developed by Turowski and Kaliszan, was initially proposed to be an in vitro tool in investigations of solutes′ skin permeability (log ***K***_p_) [34]. However, it was discovered that the retention factor obtained on this sorbent (log ***k***_KER_) is not a sufficiently good predictor of skin permeability coefficient, and it cannot be used as a sole descriptor in log ***K***_p_ models. Turowski and Kaliszan reported that this descriptor can be combined with the chromatographic retention factor obtained by immobilized artificial membrane chromatography (log ***k***_IAM_), and the results of log ***K***_p_ predictions using multiple linear regression (MLR) models satisfy (Equation (2)):

log ***K***_p_ = −6.56 + 1.92 log ***k***_IAM_ − 1.04 log ***k***_KER_ (*n* = 17, R^2^ = 0.86) (2)

Turowski and Kaliszan concluded that skin permeability increases with the lipophilicity of solutes (encoded primarily by log ***k***_IAM_) and decreases with their affinity for keratin (expressed as log ***k***_KER_). Unfortunately, the model they proposed (Equation (2)) requires two sets of chromatographic data, obtained on different stationary phases, this being the likely reason why the immobilized keratin stationary phase they proposed has never become widely popular and, to the best of our knowledge, it is not commercially available.

In this study, a novel application of immobilized keratin stationary phases developed by Turowski and Kaliszan is proposed, and chromatography on immobilized keratin sorbent is used to model compounds’ bioconcentration in aquatic organisms.

## 2. Materials and Methods

### 2.1. IAM and Immobilized Keratin Chromatography

The chromatographic retention factors for the compounds analyzed in this study (Table 1) were taken from [34]. They were obtained on: (i) an IAM.PC.MG HPLC column purchased from Regis (150 × 4.6 mm, particle diameter 12 μm, pore diameter 300 Å) with a phosphate buffer (pH 6.0), including acetonitrile (95:5 *v*/*v*) mobile phase (flow rate—1 mL min^−1^); (ii) physically immobilized keratin sorbent with pH 4.2 phosphate buffer as a mobile phase (column dimensions—125 × 4 mm; flow rate—1 mL min^−1^). The mobile phase used in keratin chromatography (pH 4.2 buffer) was selected on the basis of QSRR studies as giving the “best” relationship between log ***k***_KER_ and structural descriptors (molecular weight and dipole moment) [34].

### 2.2. Calculated Molecular Descriptors

Molecular weight (***M***_w_), heavy atom count (#***HvAt***), aromatic heavy atom count (#***ArHvAt***), fraction of sp^3^ carbons (***F***_Csp3_), rotatable bond count (*#**FRB***), hydrogen donor count (*#**HD***), hydrogen acceptor count (*#**HA***), molecular refractivity (***MR***), aqueous solubility (log ***S***), and topological polar surface area (***TPSA***) were calculated using Swiss ADME software available freely on-line [53]. The octanol–water partition coefficient (log ***K***_ow_) was predicted using EpiSuite [54]. Total counts of nitrogen and oxygen atoms (***N*** + ***O***) were calculated manually on the basis of compounds’ molecular formulas (Table 1).

### 2.3. Reference Values of Skin Permeability Coefficient (log **K**_p_) and Bioconcentration Factor (log **BCF**)

The experimentally determined values of log ***K***_p_ and log ***BCF*** are available for only some compounds within the studied group. For this reason, the models of skin permeability and bioconcentration factor, involving chromatographic and calculated descriptors, were generated and validated using log ***K***_p_ and log ***BCF*** values obtained in silico with the EpiSuite v. 4.1 software (log ***K***_p_^EPI^—DERMWIN v. 2.02 and log ***BCF***_EPI_—BCFBAF v. 3.02 modules, respectively), recommended by the US Environmental Protection Agency [54,55] and tested on sub-groups of solutes whose experimental log ***K_p_*** or log ***BCF*** values are known (log ***K***_p_^exp^, log ***BCF***_exp_) [56,57]. The estimation methodology used by DERMWIN is based on an algorithm developed by Potts [58], and the estimations provided by BCFBAF are based on methodology developed by Meylan [59] and Arnot and Gobas [3]. The values of log ***K***_p_^EPI^ and log ***BCF***_EPI_ obtained using EpiSuite are given in Table 2 and Table 3.

### 2.4. Statistical Tools

Multiple linear regression (MLR) models were generated using Statistica v. 13 by StatSoft Polska, Kraków, Poland, and this refers to the stepwise forward regression mode.

The models considered in this study were evaluated using the following procedures:

- Cross-validation was performed, with n compounds from the initial training set split into 2 subsets, one of which was used to train a new model and the remaining one to test it. After cross-validation, the RMSEP (root mean squared error of prediction) for the particular N-compound test subset was calculated as follows (Equation (3)):

(3) $$RMSEP=\sqrt{\frac{\sum_{i=1}^{N}{\left(y_{i}^{pred}-y_{i}^{ref}\right)}^{2}}{N}}$$

- Comparison of the predicted log ***K***_p_^pred^ and log ***BCF***_pred_ values (calculated for the compounds, whose experimental log ***K***_p_^exp^ and log ***BCF***_exp_ data are available) was performed, and these data were analyzed using the squared coefficient of determination (R^2^_exp_).

## 3. Results

### 3.1. Keratin vs. IAM HPLC Skin Permeability Models

In this study, we compared the log ***K***_p_ models obtained using log ***k***_IAM_ and ***TPSA*** (Equation (4)) with the models including log ***k***_KER_ as a chromatographic parameter (Equation (5)).

log ***K***_p_ = −5.61 (±0.24) + 0.68 (±0.17) log ***k***_IAM_ − 0.014 (±0.005) ***TPSA*** (*n* = 32, R^2^ = 0.63, R^2^_adj._ = 0.63, R^2^_exp_ = 0.72, F = 25.1, *p*< 0.01) (4)

log ***K***_p_ = −2.56 (±0.83) +1.74 (±0.38) log ***k***_KER_ − 0.011 (±0.008) ***M***_w_
− 0.22 (±0.11) #***ArHvAt*** − 0.014 (±0.005) ***TPSA*** (*n* = 32, R^2^ = 0.68, R^2^_adj._ = 0.63, R^2^_exp_= 0.73, F = 14.3, *p*< 0.01) (5)

**Figure 1:**
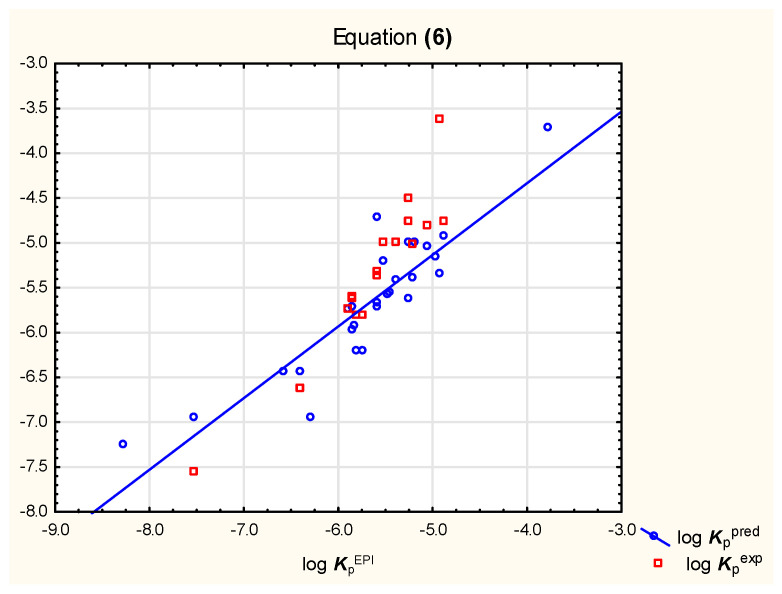
Equation (6)—predicted and experimental log ***K***_p_ vs. reference values.

**Figure 2:**
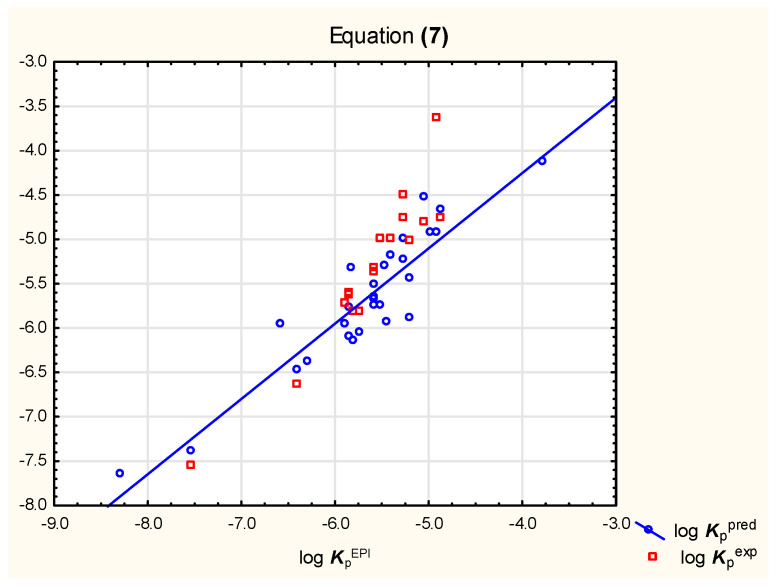
Equation (7)—predicted and experimental log ***K***_p_ vs. reference values.

It was observed that neither Equation (4), nor (5), gives satisfying results of log ***K***_p_ predictions for relatively strongly ionized solutes (compounds **14**, **16**, **23,** and **27**); when these compounds were excluded from the analysis, Equations (6) and (7) were obtained for a group of 28 neutral, basic, or weakly acidic compounds (Figure 1 and Figure 2, Table 2).

log ***K***_p_ = −5.70 + 0.81 (±0.17) log ***k***_IAM_− 0.015 (±0.004) ***TPSA*** (*n* = 28, R^2^ = 0.80, R^2^_adj._ = 0.78, R^2^_exp_ = 0.73, F = 49.7, *p*< 0.01) (6)

log ***K***_p_ = −2.73 (±0.54) +1.80 (±0.26) log ***k***_KER_ − 0.015 (±0.003) ***M***_w_ + 0.13 (±0.05) *#**HvAt*** − 0.27 (±0.07) *#**ArHvAt*** − 0.020 (±0.004) ***TPSA*** (*n* = 28, R^2^ = 0.85, R^2^_adj._ = 0.81, R^2^_exp_ = 0.79, F = 24.8, *p*< 0.01) (7)

The likely reason for such discrepancies between the predicted (Equations (4) and (5)) and reference values of log ***K***_p_ for relatively strongly ionizable compounds is that the reference model has also its limitations: it overestimates the results for very hydrophilic molecules, underestimates the values for non-hydrogen bonding solutes, and fails for extremely lipophilic compounds or solutes having a very high tendency to hydrogen bonding [60,61,62].

At this point, the group of 28 studied compounds was divided into two subsets: a training set (**1** to **20**) and a test set (**21** to **28**). Equations (8) and (9) generated for the training set, and containing the same sets of independent variables as Equations (6) and (7), are as follows (Table 2):

log ***K***_p_ = −6.09 (±0.27) + 0.94 (±0.17) log ***k***_IAM_ − 0.0073 (±0.005) ***TPSA*** (*n* = 20, R^2^ = 0.80, R^2^_adj._ = 0.78, RMSEP = 0.51, F = 34.2, *p*< 0.01) (8)

log ***K***_p_ = −1.93 (±0.54) +1.85 (±0.28) log ***k****_KER_* − 0.017 (±0.003) ***M***_w_ + 0.15 (±0.05) *#**HvAt*** − 0.37 (±0.11) *#**ArHvAt*** − 0.021 (±0.004) ***TPSA*** (*n* = 20, R^2^ = 0.87, R^2^_adj._ = 0.83, RMSEP = 0.44, F = 19.0, *p*< 0.01) (9)

### 3.2. Keratin HPLC Models of Bioconcentration Factor

**Figure 3:**
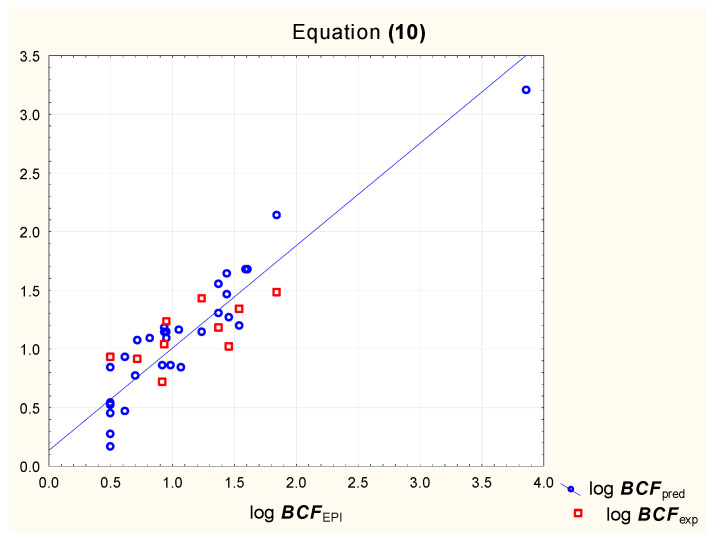
Equation (10)—predicted and experimental log ***BCF*** vs. reference values.

**Figure 4:**
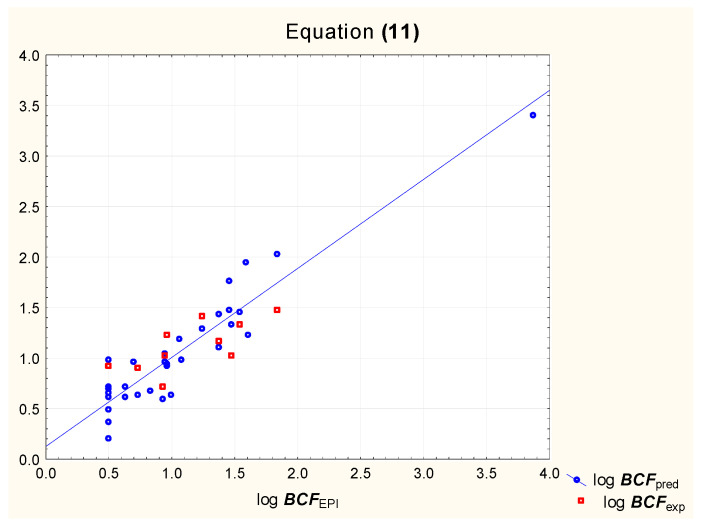
Equation (11)—predicted and experimental log ***BCF*** vs. reference values.

According to our earlier research, the bioconcentration factor log ***BCF*** can be predicted using log ***k***_IAM_ and two additional parameters: ***F***_Csp3_ and ***TPSA*** [31]. The predictive potential of Equation (10) (Figure 3) is compared to that of a model based on chromatographic retention factors obtained using immobilized keratine as a stationary phase (Equation (11), Figure 4).

log ***BCF*** = 0.79 (±0.11) + 0.62 (±0.07) log ***k***_IAM_ + 1.53 (±0.31) ***F***_Csp3_
− 0.0046 (±0.0021) ***TPSA*** (*n* = 32, R^2^ = 0.87, R^2^_adj._ = 0.86, R^2^_exp_ = 0.41, F = 63.9, *p* < 0.01) (10)

log ***BCF*** = 1.23 (±0.34) + 0.70 (±0.15) log ***k***_KER_ − 0.18 (±0.05) *#**ArHvAt*** + 0.039 (±0.006) ***MR*** − 0.017 (±0.002) ***TPSA*** (*n* = 32, R^2^ = 0.88, R^2^_adj._ = 0.86, R^2^_exp_ = 0.69, F = 50.3, *p* < 0.01) (11)

At this point, the group of 32 studied compounds was divided into two subsets: a training set (**1** to **20**) and a test set (**21** to **32**). Equations (12) and (13) generated for the training set, and containing the same sets of independent variables as Equations (10) and (11) are as follows:

log ***BCF*** = 0.46 (±0.15) + 0.75 (±0.09) log ***k***_IAM_ + 1.84 (±0.34) ***F***_Csp3_
+ 0.0010 (±0.0028) ***TPSA*** (*n* = 20, R^2^ = 0.93, R^2^_adj._ = 0.92, RMSEP = 0.36, F = 72.2, *p* < 0.01) (12)

log ***BCF*** = 1.59 (±0.57) + 0.85 (±0.20) log ***k***_KER_ − 0.22 (±0.08) *#**ArHvAt*** + 0.037 (±0.007) ***MR*** − 0.018 (±0.003) ***TPSA*** (*n* = 20, R^2^ = 0.90, R^2^_adj._ = 0.88, RMSEP = 0.23, F = 35.1, *p* < 0.01) (13)

## 4. Discussion

In our study, we investigated the possibility of using log ***k***_KER_ in skin permeability models, alongside additional descriptors that were either not considered or not available when the keratin stationary phase was originally developed. We studied correlations between log ***k***_KER_ and the key physico-chemical properties associated with compounds’ ability to cross biological barriers (Table 4) and discovered that log ***k***_KER_ encodes primarily lipophilicity (log *K*_ow_) and aqueous solubility (log ***S***), which are important factors governing the ability of compounds to cross the skin barrier, but the correlations are moderate.

Predictive models of log ***K***_p_, involving retention parameters obtained on immobilized keratin (Equations (7) and (9)), have similar (or, in fact, slightly better) statistical parameters compared to those reported for models based on IAM chromatography (Equations (6) and (8)). Log ***K***_p_ values predicted using Equation (7) are in a slightly closer agreement with experimental log ***K***_p_^exp^ data available for a subset of 18 compounds than those calculated using Equation (6). It must be noted, however, that, in the process of descriptors’ selection by forward stepwise regression, chromatographic parameters log ***k***_KER_ and log ***k***_IAM_ behave differently. Log ***k***_IAM_ (Equation (6)) is selected first, and it accounts for ca. 66% of total log ***K***_p_ variability; log ***k***_KER_ (Equation (7)) is selected second (after ***TPSA***), and it accounts for just 16% of total log ***K***_p_ variability.

The significance of log ***k***_KER_ as an independent variable is much higher in models of bioconcentration factor log ***BCF***. In Equation (11), log ***k***_KER_ is the most important independent variable, accounting for 39% of total log ***K***_p_ variability; further variables (selected as follows: ***TPSA***, ***MR,*** and #***ArHvAt***) account for 24, 18, and 7% of total log ***K***_p_ variability, respectively. In the IAM chromatography-based model of log ***BCF*** (Equation (10)), log ***k***_IAM_ accounts for 73%, and other independent variables (***F***_Csp3_ and ***TPSA***) account for 12 and 2% of total log ***K***_p_ variability, respectively. The keratin chromatographic retention-based model (11) has statistical parameters similar to those of Equation (10), derived from IAM chromatography; however, Equation (11) seems to fit the experimental data (log ***BCF***_exp_) reported for a subset of 10 compounds better than Equation (10).

## 5. Conclusions

Immobilized keratine-based chromatographic stationary phase was developed in the late 1990s to help in in vitro investigations of compounds’ transdermal absorption. A new model of a skin permeability coefficient was developed in the current study, which involves the chromatographic retention factor measured on the immobilized keratine sorbent (log ***k***_KER_) and four additional independent variables (Equation (7)). This model has slightly better statistical parameters and is in a better agreement with experimental log ***K***_p_ data than the model derived from IAM chromatography (Equation (6)); both models are applicable primarily to non-ionized compounds (with carboxylic acids removed from Equations (4) and (5)). Based on the MLR analyses conducted in this study, it was concluded that immobilized keratin chromatographic support is a moderately useful tool for skin permeability assessment. However, similarly to IAM chromatography in the past, chromatography on immobilized keratin may serve a different purpose; designed for applications in pharmacokinetic studies, it may also be of use in the realm of environmental science, in studies of compounds’ bioconcentrations in aquatic organisms.

## Figures and Tables

**Table 1:** Chromatographic retention factors and calculated physico-chemical properties of compounds **1** to **32**.

No.	Compound	log *k*_KER_	log *k*_IAM_	*M*_w_	#*HvAt*	#*ArHvAt*	*F*_Csp3_	#*FRB*	#*HA*	#*HD*	*MR*	*TPSA*	(*N* + *O*)	log *K*_ow_	log *S*
**1**	2-Cresole	−0.18	0.36	108.1	8	6	0.14	0	1	1	33.4	20.2	1	1.95	−2.29
**2**	2-Naphtol	0.88	1.25	144.2	11	10	0	0	1	1	46.0	20.2	1	2.70	−3.11
**3**	3-Cresole	−0.22	0.36	108.1	8	6	0.14	0	1	1	33.4	20.2	1	1.96	−2.30
**4**	3-Nitrophenol	0.24	0.60	139.1	10	6	0	1	3	1	37.3	66.1	4	2.00	−2.34
**5**	4-Bromophenol	0.34	1.00	173.0	8	6	0	0	1	1	36.2	20.2	1	2.59	−3.10
**6**	4-Chlorophenol	0.27	0.73	128.6	8	6	0	0	1	1	33.5	20.2	1	2.39	−2.70
**7**	4-Cresole	−0.08	0.42	108.1	8	6	0.14	0	1	1	33.4	20.2	1	1.94	−2.29
**8**	4-Ethylphenol	−0.25	0.76	122.2	9	6	0.25	1	1	1	38.2	20.2	1	2.58	−2.65
**9**	4-Nitrophenol	0.19	0.60	139.1	10	6	0	1	3	1	37.3	66.1	4	1.91	−2.28
**10**	Baclofen	−0.33	−0.73	213.7	14	6	0.3	4	3	2	55.3	63.3	3	−0.96	−0.61
**11**	Chlorocresole	0.68	1.18	142.6	9	6	0.14	0	1	1	38.4	20.2	1	2.70	−3.09
**12**	Methylparaben	0.04	0.52	152.2	11	6	0.12	2	3	1	39.7	46.5	3	1.96	−2.29
**13**	Phenol	−0.27	0.37	94.1	7	6	0	0	1	1	28.5	20.2	1	1.46	−1.98
**14**	Phenylalanine	−0.20	−0.65	165.2	12	6	0.22	3	3	2	45.5	63.3	3	−1.44	−0.08
**15**	Resorcinol	−0.38	−0.14	110.1	8	6	0	0	2	2	30.5	40.5	2	0.80	−1.58
**16**	Salcylic acid	−0.06	−0.58	138.1	10	6	0	1	3	2	35.4	57.5	3	2.26	−2.50
**17**	Thymol	0.52	1.34	150.2	11	6	0.4	1	1	1	48.0	20.2	1	3.30	−3.19
**18**	1,2,3-tris(1-methylethyl)benzene	0.75	2.43	204.4	15	6	0.6	3	0	0	70.2	0.0	0	6.36	−4.54
**19**	1,4-dinitrobenzene	0.45	0.16	168.1	12	6	0	2	4	0	44.1	91.6	6	1.46	−2.04
**20**	3-(trifluoromethyl)phenol	0.19	1.23	162.1	11	6	0.14	1	4	1	33.5	20.2	1	2.95	−3.04
**21**	4-cyanophenol	−0.05	0.77	119.1	9	6	0	0	2	1	33.2	44.0	2	1.60	−2.08
**22**	4-iodophenol	0.80	1.59	220.0	8	6	0	0	1	1	41.2	20.2	1	2.91	−3.59
**23**	4-nitrobenzoic acid	−0.23	−0.23	167.1	12	6	0	2	4	1	42.2	83.1	5	1.89	−2.30
**24**	Anizole	−0.09	0.31	108.1	8	6	0.14	1	1	0	32.9	9.2	1	2.11	−2.33
**25**	Benzamide	−0.04	−0.10	121.1	9	6	0	1	1	1	34.5	43.1	2	0.64	−1.42
**26**	benzene	−0.27	0.09	78.1	6	6	0	0	0	0	26.4	0.0	0	2.13	−2.41
**27**	benzoic acid	−0.21	−0.74	122.1	9	6	0	1	2	1	33.4	37.3	2	1.87	−2.20
**28**	Benzonitrile	0.02	0.15	103.1	8	6	0	0	1	0	31.2	23.8	1	1.56	−2.02
**29**	caffeine	0.08	−0.40	194.2	14	9	0.38	0	3	0	52.0	61.8	6	−0.07	−1.48
**30**	Chlorobenzene	0.13	0.66	112.6	7	6	0	0	0	0	31.5	0.0	0	2.84	−2.96
**31**	Indazole	0.23	0.71	118.1	9	9	0	0	1	1	36.1	28.7	2	1.77	−2.72
**32**	Toluene	−0.05	0.44	92.1	7	6	0.14	0	0	0	31.4	0.0	0	2.73	−2.77

**Table 2:** Reference (EPI), predicted, and experimental values of log ***K***_p_.

	log *K*_p_^EPI^	Equation (6)	Equation (7)	Equation (8)	Equation (9)	log *K*_p_^exp^
2-Cresole	−5.58	−5.71	−5.67	−5.89	−5.57	−5.36
2-Naphtol	−5.26	−4.99	−5.00	−5.05	−5.26	−4.76
3-Cresole	−5.57	−5.71	−5.75	−5.89	−5.65	−5.37
3-Nitrophenol	−5.73	−6.22	−6.06	−6.01	−5.98	−5.81
4-Bromophenol	−5.52	−5.20	−5.74	−5.30	−5.74	−5.00
4-Chlorophenol	−5.39	−5.42	−5.18	−5.55	−5.09	−5.00
4-Cresole	−5.58	−5.67	−5.50	−5.84	−5.39	−5.31
4-Ethylphenol	−5.21	−5.39	−5.88	−5.52	−5.80	−5.01
4-Nitrophenol	−5.79	−6.22	−6.15	−6.01	−6.08	−5.81
Baclofen	−8.28	−7.25	−7.66	−7.23	−7.69	
Chlorocresole	−5.05	−5.05	−4.53	−5.12	−4.43	−4.82
Methylparaben	−5.84	−5.98	−6.09	−5.94	−6.02	−5.63
Phenol	−5.84	−5.71	−5.76	−5.89	−5.65	−5.61
Resorcinol	−6.40	−6.43	−6.48	−6.52	−6.40	−6.63
Thymol	−4.87	−4.92	−4.66	−4.97	−4.56	−4.77
1,2,3-tris(1-methylethyl)benzene	−3.78	−3.73	−4.13	−3.80	−4.06	
1,4-dinitrobenzene	−6.29	−6.96	−6.38	−6.61	−6.32	
3-(trifluoromethyl)phenol	−5.19	−5.01	−5.44	−5.07	−5.38	
4-cyanophenol	−5.89	−5.74	−5.96	−5.68	−5.86	−5.73
4-iodophenol	−5.58	−4.71	−5.64	−4.74	−5.70	
Anizole	−5.46	−5.59	−5.29	−5.86	−5.18	
Benzamide	−6.58	−6.43	−5.96	−6.50	−5.86	
Benzene	−5.26	−5.63	−5.22	−6.00	−5.09	−4.51
Benzonitrile	−5.82	−5.94	−5.31	−6.12	−5.19	
Caffeine	−7.53	−6.96	−7.38	−6.91	−7.66	−7.56
Chlorobenzene	−4.97	−5.17	−4.92	−5.47	−4.81	
Indazole	−5.44	−5.56	−5.94	−5.63	−6.11	
Toluene	−4.92	−5.35	−4.92	−5.68	−4.78	−3.64

**Table 3:** Reference, predicted, and experimental values of log ***BCF***.

	log *BCF*_EPI_	Equation (10)	Equation (11)	Equation (12)	Equation (13)	log *BCF*_exp_
2-Cresole	0.95	1.13	0.97	1.01	0.98	1.03
2-Naphtol	1.45	1.47	1.48	1.43	1.45	
3-Cresole	0.96	1.13	0.94	1.01	0.94	1.23
3-Nitrophenol	0.99	0.85	0.64	0.98	0.67	
4-Bromophenol	1.38	1.31	1.44	1.23	1.52	1.17
4-Chlorophenol	1.24	1.14	1.29	1.03	1.37	1.42
4-Cresole	0.95	1.17	1.04	1.06	1.06	
4-Ethylphenol	1.37	1.55	1.11	1.52	1.10	
4-Nitrophenol	0.93	0.85	0.60	0.98	0.63	0.71
Baclofen	0.50	0.51	0.99	0.53	0.90	
Chlorocresole	1.45	1.64	1.77	1.63	1.90	
Methylparaben	0.96	1.08	0.93	1.12	0.94	
Phenol	0.63	0.92	0.71	0.76	0.71	
Phenylalanine	0.50	0.44	0.70	0.44	0.65	
Resorcinol	0.50	0.51	0.37	0.40	0.34	
Salcylic acid	0.50	0.17	0.50	0.09	0.50	
Thymol	1.84	2.13	2.03	2.23	2.12	1.48
1,2,3-tris(1-methylethyl)benzene	3.86	3.20	3.41	3.40	3.49	
1,4-dinitrobenzene	0.63	0.46	0.62	0.67	0.65	
3-(trifluoromethyl)phenol	1.61	1.67	1.23	1.67	1.30	
4-cyanophenol	0.72	1.06	0.65	1.09	0.66	0.91
4-iodophenol	1.59	1.67	1.96	1.68	2.10	
4-nitrobenzoic acid	0.50	0.27	0.21	0.37	0.15	
Anizole	1.06	1.15	1.20	0.96	1.23	
Benzamide	0.50	0.53	0.72	0.43	0.73	
Benzene	1.07	0.84	0.98	0.53	1.00	
Benzoic acid	0.50	0.16	0.66	−0.06	0.65	0.93
Benzonitrile	0.70	0.77	0.96	0.60	1.00	
Caffeine	0.50	0.84	0.63	0.93	0.48	
Chlorobenzene	1.54	1.19	1.45	0.95	1.52	1.34
Indazole	0.83	1.09	0.67	1.03	0.61	
Toluene	1.47	1.27	1.33	1.05	1.37	1.02

**Table 4:** Correlations ^®^ between chromatographic and calculated parameters (*n*= 32).

	log *k*_KER_	log *k*_IAM_	*M*_w_	*#HvAt*	#*ArHvAt*	*F*_Csp3_	#*FRB*	#*HA*	#*HD*	*MR*	*TPSA*	log *K*_ow_	log *S*
log ***k***_KER_	1.00	0.75	0.48	0.26	0.33	0.15	−0.07	−0.15	−0.25	0.45	−0.16	0.57	−0.67
log ***k***_IAM_	**0.75**	1.00	0.20	0.00	0.06	0.26	−0.19	−0.42	−0.31	0.26	−0.54	0.81	−0.85
***M***_w_	0.48	0.20	1.00	0.77	0.12	0.43	0.57	0.43	0.18	0.80	0.39	0.01	−0.09
#***HvAt***	0.26	0.00	0.77	1.00	0.25	0.62	0.75	0.56	0.13	0.89	0.54	−0.08	0.11
#***ArHvAt***	0.33	0.06	0.12	0.25	1.00	0.03	−0.24	−0.02	−0.09	0.24	0.02	−0.09	−0.05
***F***_Csp3_	0.15	0.26	0.43	0.62	0.03	1.00	0.46	−0.09	−0.12	0.75	−0.16	0.21	−0.13
#***FRB***	−0.07	−0.19	0.57	0.75	−0.24	0.46	1.00	0.49	0.25	0.66	0.48	−0.18	0.30
#***HA***	−0.15	−0.42	0.43	0.56	−0.02	−0.09	0.49	1.00	0.38	0.18	0.87	−0.45	0.45
#***HD***	−0.25	−0.31	0.18	0.13	−0.09	−0.12	0.25	0.38	1.00	0.00	0.37	−0.42	0.40
***MR***	0.45	0.26	0.80	0.89	0.24	0.75	0.66	0.18	0.00	1.00	0.23	0.16	−0.14
***TPSA***	−0.16	−0.54	0.39	0.54	0.02	−0.16	0.48	0.87	0.37	0.23	1.00	−0.57	0.57
log ***K***_ow_	**0.57**	0.81	0.01	−0.08	−0.09	0.21	−0.18	−0.45	−0.42	0.16	−0.57	1.00	−0.96
log ***S***	**−0.67**	−0.85	−0.09	0.11	−0.05	−0.13	0.30	0.45	0.40	−0.14	0.57	−0.96	1.00

## Data Availability

Data generated in this study can be found in this manuscript.
